# Measuring Situational Cognitive Performance in the Wild: A Psychometric Evaluation of Three Brief Smartphone-Based Test Procedures

**DOI:** 10.1177/10731911231213845

**Published:** 2023-12-14

**Authors:** Johanna Perzl, Elisabeth Maria Riedl, Joachim Thomas

**Affiliations:** 1 Catholic University of Eichstätt-Ingolstadt, Germany; 2 University of Würzburg, Germany

**Keywords:** cognitive performance, ambulatory assessment, smartphone, sustained attention to response task, psychomotor vigilance task, digit symbol substitution task, situational fluctuations

## Abstract

Mobile devices provide new opportunities to draw conclusions about cognitive performance in everyday situations. To gain insights into cognitive performance patterns in healthy adult populations, we adapted three established cognitive tests for smartphone use: the Digit Symbol Substitution Task (DSST), Sustained Attention to Response Task (SART), and Psychomotor Vigilance Task (PVT). To increase their feasibility for ambulatory assessment, we identified the minimum measurement durations that provide reliable and valid state measures of cognitive performance. Over 2 weeks, 46 participants performed each test once per day at random times, along with self-reports (e.g., on concentration, mood, and mental demands). The validity and reliability of change are promising for the 30-second PVT and 90-second DSST and SART. The DSST and SART provide fruitful outcomes for ambulatory field studies linked to mood, stress, and mental demands. We provide digital versions of the adapted DSST and SART online for free.

Traditionally, cognitive testing is conducted as a single measurement by trained technicians in standardized settings and does not consider within-person variation in cognitive functioning (Sliwinski et al., 2018). This approach is inconsistent with latent state-trait theory, according to which the measurement of a variable should consider both stable and variable components (Steyer et al., 1999). Furthermore, typical cognitive performance in everyday situations cannot be equated with optimal attentional performance measured under artificial and standardized laboratory conditions. Under these conditions, the influence of social context on cognitive functioning and the interaction of the individual with the environment can never be fully pictured (Moore et al., 2017), which diminishes ecological validity (Allard et al., 2014; Timmers et al., 2014). Repeated ambulatory cognitive assessment, however, may guarantee ecological validity by measuring momentary attention in real-life contexts (Hoc, 2001; Reis, 2012; Timmers et al., 2014; Trull & Ebner-Priemer, 2013). This adds value by providing insights into cognitive trajectories or processes and situational determinants of cognitive performance (Moore et al., 2017). However, ambulatory assessment studies often rely exclusively on self-report measures, which risks overestimating the true association due to common method variance (Campbell & Fiske, 1959; Podsakoff et al., 2003), especially when facing abstract constructs, such as cognitive performance (Belenky et al., 2003; Bermudez et al., 2016; Cote & Buckley, 1987; Dorrian et al., 2003).

There has been a repeated call to integrate objective data in occupational research (e.g., Sonnentag et al., 2017). In particular, cognitive performance is crucial to guarantee optimal performance, safety, and well-being at work (Akerstedt & Wright, 2009; Brown, 1994; J. Chung et al., 2015; Harrison & Horne, 2000) and is affected by work characteristics (Qian et al., 2015; Rich et al., 2010). However, the few studies that have applied cognitive tests in real-life occupational settings have either been conducted in a clinical context—for example, linked to alcohol consumption (A. Jones et al., 2018; Tiplady et al., 2009)—or the participants examined (e.g., shift workers, firefighters, pilots) were employed in very specific time-constrained work environments (e.g., Gander et al., 2013; Matsangas & Shattuck, 2020; Patterson et al., 2019; Petrilli et al., 2006; Stout et al., 2021), focusing on performance decline due to shift work and suboptimal sleep behavior without considering fatigue caused by job demands. To date, the use of objective cognitive measures in ambulatory assessment studies with healthy adults is rare (for an exception see e.g., Daniëls et al., 2020), even though new technologies offer various options to combine subjective measures with objective or physiological measures (Moore et al., 2017) to picture dynamic psychological processes in a person’s natural environment (Trull & Ebner-Priemer, 2013).

Measuring cognitive performance using a smartphone-based approach by adapting existing computerized tests successfully applied in traditional cognitive studies is an option for ambulatory cognitive assessment (Calamia, 2019). The challenge for objective ambulatory assessment, however, is to keep the measurements as short as possible while still ensuring measurement quality. As study participants are surveyed repeatedly, the primary goal is to minimize the participant burden, maintain compliance, and reduce the risk of external distraction impairing cognitive performance (Sliwinski et al., 2018). However, very little is known about the *shortest* measurement durations of specific cognitive tasks that still provide sufficient measurement quality. The measurement duration of the cognitive tests used in former daily diary studies was either very long (e.g., A. Jones et al., 2018; Tiplady et al., 2009) or chosen based on practical assumptions rather than on empirical evidence (e.g., T. Chung et al., 2020; Lam et al., 2022; Verhagen et al., 2019). There is a need to provide psychometric evidence to encourage researchers to supplement their purely subjective questionnaires with objective performance tests, not only for the validity and reliability of cognitive performance tests in everyday life but also for their sensitivity to systematic within-person changes in cognitive performance, especially over short periods (Sliwinski et al., 2018).

Our major aim is to evaluate whether, how, and when three established cognitive test procedures can be fruitfully applied with smartphones for repeated ambulatory assessments in healthy adult populations to gain insights into cognitive performance patterns. By answering the research question about the *shortest* measurement duration of each test that provides reliable and valid smartphone-based state measures of typical cognitive performance, we pave the way to increase the feasibility of the ambulatory use of three established cognitive test procedures. We further identify associations of their objective outcomes with contextual time-variant variables to give practitioner recommendations for an evidence-based choice among specific tests for different research domains. By providing the digital version of the test procedures used in reliable and valid measurement durations online for free, we help researchers easily implement them in their smartphone-based ambulatory assessment studies.

## Theoretical Background

We focus on three tasks that cover different aspects of cognitive performance to answer our research questions: a matching task measuring information processing speed adapted from the Digit Symbol Substitution Task (DSST), a reaction time task measuring alertness adapted from the Psychomotor Vigilance Task (PVT), and a Go-NoGo task measuring cognitive inhibition adapted from the Sustained Attention to Response Task (SART).

### Three Established Tests to Measure Objective Cognitive Performance

#### Digit Symbol Substitution Task

We selected the DSST for our study because DSST performance can be considered a measure of complex attention (Lezak, 1995) and therefore be interpreted as an indicator of general cognitive functioning (Dickinson et al., 2007; Salthouse, 1996) that has low specificity (Amaresha et al., 2014; Sandry et al., 2021) but is highly sensitive to acute and chronic cognitive change (Jaeger, 2018; Sandry et al., 2021). These characteristics make it a promising test procedure for monitoring momentary cognitive functioning (Jaeger, 2018) in healthy samples working in various occupational domains. In addition, participants in ambulatory assessment studies rated the remote DSST as feasible and acceptable (John et al., 2021; van Oirschot et al., 2020). Participants have reported that the DSST is easy to use and is pleasant and doable (Daniëls et al., 2020; Verhagen et al., 2019). Furthermore, participants were motivated to perform well (Daniëls et al., 2020).

The DSST is a matching task that requires the individual to match random digits to the corresponding symbols based on a provided key (Jaeger, 2018). Originally, the DSST was conducted as a 90- to 120-second paper and pencil test. The traditional test score is the total number of correctly matched symbols within the predefined measurement time. The outcomes usually used are the number of correct responses (T. Chung et al., 2020; Lam et al., 2022; Suffoletto et al., 2017), the percentage of correct trials, namely, accuracy (Daniëls et al., 2020; Verhagen et al., 2019), and the mean reaction time (T. Chung et al., 2020). When monitoring cognitive change, practice effects within and between days need to be accounted for even if differing versions of the test are presented (Beres & Baron, 1981; van Oirschot et al., 2020; Verhagen et al., 2019).

To date, the DSST and similar tests have mostly been applied to samples of elderly individuals (Brouillette et al., 2013) and clinical patients (Allen et al., 2021; T. Chung et al., 2020; Suffoletto et al., 2017; van Oirschot et al., 2020), and in some cases, even outside the laboratory in ambulatory assessment designs (e.g., T. Chung et al., 2020; Lam et al., 2022; Suffoletto et al., 2017; van Oirschot et al., 2020). In two studies, healthy adults were included as the control group (Lam et al., 2022; van Oirschot et al., 2020). Only two studies have been conducted that focused mainly on a healthy adult sample: Daniëls et al. (2020) and Verhagen et al. (2019). These studies evaluated the validity of a 30-second smartphone-based DSST by contextualizing cognitive performance with intrapersonal and situational factors in everyday life. Distraction, social context, location, and mood were found to be relevant for cognitive performance (Daniëls et al., 2020; Verhagen et al., 2019).

While the measurement durations of the digital DSST in the studies mentioned ranged from 30 seconds (Daniëls et al., 2020; Verhagen et al., 2019) to 2 minutes (Brouillette et al., 2013), assessment density ranged from an assessment every 3 days (Lam et al., 2022; van Oirschot et al., 2020) to an hourly assessment (Suffoletto et al., 2017). Overall, there was promising support for both concurrent and construct validity (Allen et al., 2021; Brouillette et al., 2013; T. Chung et al., 2020; Lam et al., 2022; Suffoletto et al., 2017; van Oirschot et al., 2020) and convincing reliability (Brouillette et al., 2013; John et al., 2021; Lam et al., 2022; van Oirschot et al., 2020) among the measurement durations of smartphone-based DSST versions for clinical patient or elderly individual samples, while healthy adult samples remain underrepresented.

In summary, the smartphone-based DSST seems to differ widely in its applied measurement duration, hindering the comparability across studies. Furthermore, the DSST has mostly been applied in the clinical context to date (e.g., T. Chung et al., 2020; Lam et al., 2022), restricting generalization to a healthy occupational population. Information is lacking concerning the minimum measurement duration that provides reliable and valid information about cognitive functioning in a healthy adult sample, as the overarching aim should be to prevent unnecessary burdening of the participants.

#### Psychomotor Vigilance Task

We further chose the PVT for our study as it depicts real-world risks, especially in the context of tasks that require work-paced or timely responses, such as industrial or transportation tasks (Basner & Dinges, 2011; Dinges, 1995; Philip & Akerstedt, 2006). Sleep deprivation and fatigue are reflected in failures of vigilant attention (Lim & Dinges, 2008); thus, the PVT is commonly used by sleep clinicians. However, fatigue at work can be caused not only by sleep loss but also by work characteristics, such as sustained mental workload or long working hours (Lim et al., 2010; Peng et al., 2021). Furthermore, fatigue can have serious consequences in several workplaces due to its association with high accident risk and low performance (Basner & Dinges, 2011; Macchi et al., 2002; Peng et al., 2021).

The PVT is a simple visual reaction time task intended to be an indicator of sustained attention, information processing speed, cognitive fatigue, and alertness (Basner & Dinges, 2011; Dinges & Powell, 1985; Doran et al., 2001; Price et al., 2017; van Dongen et al., 2003). A black screen is initially presented. The participant is asked to touch the screen as soon as a checkerboard pattern appears, which is presented at random interstimulus intervals. Among published studies, PVT performance outcomes vary widely, whereas metrics based on response speed and lapses, defined as reaction times that exceed a certain threshold, seem to have the highest sensitivity to sleep loss (Basner & Dinges, 2011).

Since more than a decade, an effort has been made to validate the well-established PVT for ambulatory use with handheld devices and short measurement durations (Lamond et al., 2005; Loh et al., 2004). Most of the validation studies, however, were conducted in the laboratory (Basner & Rubinstein, 2011; Brunet et al., 2017; Grant et al., 2017; Honn et al., 2015; Lamond et al., 2005, 2008; Loh et al., 2004; Roach et al., 2006), which prevents generalization due to a lack of ecological validity. Currently, the shortest validated measurement duration for the PVT in these studies—and for technical devices such as tablets, smartphones, or wrist-worn PVT devices—is 3 minutes (Basner et al., 2011; Basner & Rubinstein, 2011; Brunet et al., 2017; Grant et al., 2017; Matsangas et al., 2017), which still presents a risk in terms of compliance with repeated ambulatory assessments, especially in the occupational context. In regard to validation studies outside the laboratory, M. J. Jones et al. (2018) applied the test in the real-life natural context of female basketball players but unfortunately did not find validity evidence for the 3-minute ambulatory PVT presented on iPads. Price et al. (2017), however, indicated that valid and reliable daily measures of cognitive fatigue can be obtained even with a very short PVT test duration comprising 20 trials in a healthy sample.

While Qian et al. (2015) showed a performance decline in the 20-minute PVT due to heat stress and Peng et al. (2021) showed a mediating effect of fatigue on the association of workplace characteristics and accident risk, little is known about whether and how fatigue caused by work demands manifests in PVT performance. Thus, it remains unclear whether the ambulatory application of the PVT in occupational groups other than those working in time-constrained work environments is fruitful for future research. Furthermore, the common measurement durations remain too time-consuming for ambulatory assessment studies.

#### Sustained Attention to Response Task

The SART is the third cognitive test applied in our study. We chose this Go-NoGo task because we consider its outcomes relevant in the occupational context, especially for tasks or situations where response inhibition is crucial to avoid errors or accidents (Wilson et al., 2018). The SART requires effortful attention (Grier et al., 2003), and its outcomes are significantly associated with general cognitive failures (Smilek et al., 2010). Furthermore, some work characteristics are associated with attentional degradation measured by the SART (Qian et al., 2015).

The SART was constructed to measure everyday attention failures and provides several indicators of sustained attention (Robertson et al., 1997). During the task, random digits from 1 to 9 appear on the screen. Participants are asked to respond each time any new digit apart from the number 3 appears. The original version has a test duration of 4.3 minutes, which corresponds to 25 passes of digits 1 to 9. The most prominent outcome is the number of commission errors, which represents the number of responses given in a NoGo trial. This outcome can be seen as an indicator of response accuracy, controlled attention (Manly et al., 2003), and response inhibition (Johnson et al., 2007). In addition, further outcomes can be considered, such as the mean reaction time or the standard deviation of reaction time, which provides information about the stability of the response style. To minimize the influence of the response strategy, Schmidt-Atzert et al. (2004) recommend considering the reaction times of correct trials.

The SART has been applied in some recovery studies. For example, SART performance improvement was observed by Pasanen et al. (2018) following nature walks and restoration-enhancement tasks. To our knowledge, the study by Riedl et al. (2023) is the only one in which the SART was provided ambulatorily on smartphones in the work context and used with a measurement duration of under 3 minutes. In this study, it was shown that live-streaming break interventions can have positive effects on SART performance after a work break. One reason for occupational field researchers not using an ambulatory SART in their studies might be the relatively long traditional measurement duration as well as a lack of information concerning the data quality of shortened versions of the task presented on handheld devices, which highlights the need for empirical evidence concerning the reliability and validity of brief smartphone-based versions of this cognitive test.

### Objective Cognitive Performance and Subjective Concentration

One fundamental requirement to perform a cognitive task is concentration, which enables the individual to ignore distractions and focus on the task (Moran, 2012). According to Matlin (2009), attention can also be defined as the concentration of mental activity. Concentration as the conscious decision of an individual to invest mental effort into an aspect of the current situation can be interpreted as one dimension of attention (Moran, 2012). Therefore, concentration can be assumed to be closely related to cognitive performance.

### Time-Variant Variables Related to Cognitive Performance

Previous studies devoted to the within-person reliability of various brief cognitive test procedures (e.g., Sliwinski et al., 2018) lack information on whether satisfying reliability coefficients are meaningful for identifying the impact of related time-variant variables, such as mood, fatigue, or stress on cognitive performance. As we focus on healthy adult populations that are largely employed, we further consider the contextual variable of mental demands to be relevant.

#### Cognitive Performance and Valence

Previous research has shown that emotions and feelings can affect cognitive processes, such as perception and attention, which play a crucial role in the first stages of information processing. Emotions can be characterized dimensionally, for example, by valence-based theories differing between positive and negative emotions (LeBlanc et al., 2015). According to the broaden-and-build theory (Fredrickson, 2001), positive emotions broaden individuals’ scope of attention and cognition. Consistent with this theory, Fredrickson and Branigan (2005) demonstrated that positive emotions lead to better scores in a visual processing task. In addition, medical students in the positive-affect condition were more efficient in an anagram task than participants in the control group, whereas their accuracy did not significantly differ (Isen et al., 1991). Furthermore, positive emotions of athletes were significantly associated with performance-relevant focus and therefore promoted concentration and performance (Vast et al., 2010). When participants reported increased negative affect in the study of Brose et al. (2012), they showed poorer working memory performance. In further studies by Brinker et al. (2013), negative mood significantly predicted correct hits in cognitive tests when the cognitive load was low and errors of inhibition when the cognitive load was low or high. Furthermore, in an ambulatory assessment study by Verhagen et al. (2019), mood correlated with cognitive variation measured eight times per day using a 30-second smartphone-based DSST in a sample of healthy adults. Overall, the broaden-and-build theory and previous empirical findings lead to the assumption that cognitive performance is positively associated with momentary valence.

#### Cognitive Performance and Arousal

The level of arousal can also impact cognitive performance. Regarding subjective arousal states, two dimensions can be distinguished: energetic arousal (energy vs. fatigue) and tense arousal (tension vs. calmness; Thayer, 1990, 1997)

##### Energetic Arousal

Energetic arousal is typically seen as a performance facilitator (G. Matthews & Westerman, 1994) since the subjective energy level may indicate the extent of available attentional resources (Hirst & Kalmar, 1987; G. Matthews & Davies, 2001). This effect becomes especially evident when task difficulty is high (G. Matthews et al., 1990). Overall, this leads to the assumption that when individuals subjectively feel energetic, more resources are available, and therefore, better cognitive performance is shown.

##### Tense Arousal

In contrast, according to the attention-depletion hypothesis, tense arousal depletes attentional resources (Sliwinski et al., 2006). It is assumed that more resources are available when the stress level is low than when it is high, predicting a negative within-person correlation between experienced stress and cognitive performance. Resource depletion can impair cognitive processing when it is effortful and therefore depends on available resources (Kahneman, 1973; Oei et al., 2006). Eysenck et al. (2007) state in Attentional Control Theory (ACT) that situational stress is associated with impaired attentional control, especially when the task is highly demanding. According to the ACT, situational stress mainly affects the central executive functions inhibition and shifting of attention and, to a lesser extent, memory updating (Miyake et al., 2000).

In line with the assumptions concerning tense arousal, in a study by Sänger et al. (2014), the error rate of stressed participants was increased in comparison with those in the control group, especially when top-down control was necessary to solve luminance-detection tasks. Shields et al. (2016) conducted a meta-analysis and determined that a negative stress effect was evident for working memory tasks, cognitive flexibility tasks, and cognitive inhibition tasks, such as the SART, or simple reaction time tasks. Sliwinski et al. (2006) revealed that situational variability in stress-predicted attentional performance in a working memory task within persons, with slower reaction times detected on stressful days. However, stress effects did not become evident when the tasks were performed in simple versions imposing lower working memory demands.

Stress-related performance effects have also been found in occupational samples, where stress was found to be negatively associated with working memory and work performance in health professionals (Allan et al., 2014; Cheung & Au, 2011; Harvey et al., 2012; LeBlanc, 2009; Pottier et al., 2013) and special operations soldiers (Morgan et al., 2006). Furthermore, stress is a very relevant outcome in the occupational research context, as work stress and general stress correlate significantly with occupational cognitive failures (Hussain et al., 2019; Wadsworth et al., 2003), which can in turn translate into workplace accidents or injuries and patient safety incidents (Day et al., 2012; Park & Kim, 2013; Wadsworth et al., 2003). Overall, the theoretical assumptions and previous empirical findings indicate that performance in cognitive tasks is enhanced when tense arousal is lower, meaning that individuals feel calmer (Thayer, 1990, 1997).

#### Cognitive Performance and Mental Demands

In the occupational context, it is particularly relevant to consider demands with respect to cognitive performance. Young and Stanton (2002) state in their Malleable Attentional Resources Theory (MART) that cognitive performance decreases as a consequence of cognitive underload. In the frame of their theory, the authors point out that attentional capacity can temporarily change according to the mental demands that an individual is facing. For example, low mental demands can lead to a reduced attentional capacity and a performance deficit in subsidiary cognitive tasks due to decreasing cognitive resources. To conclude, according to the MART, low mental demands can be considered detrimental to cognitive performance. This observation is further supported by the results of Liao and Moray (1993), showing that the information-processing time when completing cognitive tests is faster when facing increased time pressure, which might be linked to an increased attentional capacity. Furthermore, De Grip et al. (2008) found a cognitive decline in highly educated employees who worked in unchallenging jobs for which they were overqualified. The theoretical conclusions and previous empirical findings suggest a positive relationship between cognitive performance and cognitive demands.

### The Present Study

Although there is a great need to draw solid conclusions about the influence of occupational psychosocial factors on cognitive performance in healthy samples, occupational studies conducting ambulatory cognitive assessment are strongly underrepresented. This lack of research may be because most well-established cognitive test procedures have rarely been validated for efficient ambulatory use in healthy samples. Due to the traditional measurement procedures in the laboratory, the application of cognitive tests might often be assigned to long measurement times, which is particularly problematic in repeated surveys conceived at the workplace. This effect leads to our first two research questions:

**Research Question 1 (RQ1):** What is the shortest measurement duration of the smartphone-based DSST, PVT, and SART that provides *reliable* measures of within-person change in cognitive performance?
**Research Question 2 (RQ2):** Do the shortened reliable smartphone-based DSST, PVT, and SART produce *valid* measures of cognitive performance?

Furthermore, we aim to determine whether the brief test versions are sensitive to relationships between cognitive performance and related time-variant variables such as valence, energetic arousal, calmness, and preceding mental demands (Sliwinski et al., 2018). Therefore, we aim to answer our third research question:

**Research Question 3 (RQ3):** Are the brief smartphone-based DSST, PVT, and SART significantly associated with related time-variant variables?

By answering this question, we intend to provide an outlook on potentially fruitful fields of application.

## Method

### Sample and Procedure

The study was conducted among first-year psychology students at a German university. The study design covered two full weeks, including 14 days from Monday to Sunday. At the beginning of the study period, the participants answered a short one-time smartphone survey including demographic variables and demos of the three cognitive tests. Each day, at three semirandom time points, the students received a smartphone alert that announced a smartphone questionnaire including a small set of subjective items and, subsequently, one of three short cognitive tests. The cognitive tests were presented one at a time to keep momentary measurement durations short and to avoid the influence of mental fatigue on subsequent test performance (Kato et al., 2009). Each cognitive test was presented once a day. One alert appeared randomly between 9 a.m. and 1 p.m., one between 1 p.m. and 5 p.m. and one between 5 p.m. and 9 p.m. Alarms could be postponed for up to 90 minutes and rejected. There was a minimum break of 2 hours between the alarms.

As our research questions focus on Level 1 effects, we aimed for a minimum sample size of *N_2_* = 40 (Arend & Schäfer, 2019). Forty-seven students registered for the study. One was excluded because the criterion of at least one completed measurement per cognitive test was not fulfilled. Five students were male, 40 were female, and one did not provide any demographic information. The participating students were aged between 18 and 42 years (*M* = 20.27; *SD* = 3.99). The remaining 46 students provided 1,706 valid daily measurements within the foreseen 14-day assessment interval, covering 575 valid data sets for the PVT and 566 and 559 valid data sets for the DSST and SART, respectively, corresponding to a compliance rate of 88.30%. Participating students were credited with experimental subject hours depending on their compliance. The ethical aspects of the study were evaluated and approved by the ethics committee of the Catholic University of Eichstätt-Ingolstadt (approval no. 088–2021). General Data Protection Regulation guidelines were followed, and informed consent was obtained from all participants. The participants were informed that they could withdraw their consent anytime during the assessment period without risking any negative impact on their performance evaluations or relations with their professors.

### Measures

Unless otherwise stated, variables were measured on a seven-point Likert-type scale from *strongly disagree* (1) to *strongly agree* (7). In the interest of parsimony in repeated measures, we used a single-item scale for subjective concentration, which, according to R. A. Matthews et al. (2022), does not raise concerns about unreasonable losses of psychometric goodness criteria. As this study was a part of a larger research project, further variables concerning sleep, memory, and preceding concentration difficulties were assessed in the questionnaires. However, these additional variables were not included in our analyses and therefore will not be described in more detail.

#### Subjective Concentration

We measured difficulty in maintaining focused attention performance in the one-time smartphone survey at the beginning of the study period with the German version of the Attention and Performance Self-Assessment by Bankstahl and Görtelmeyer (2013). An exemplary item is “In the last 4 weeks, I was only able to concentrate for a very short period of time.” Momentary subjective concentration was recorded situationally with the item “At the moment I can concentrate very well” (Jacobs, 2014).

#### Subjective Valence and Arousal

Momentary mood was assessed with the short scale of Wilhelm and Schoebi (2007) covering the three basic mood dimensions with two bipolar items each: valence (discontent vs. content and unwell vs. well), calmness (agitated vs. calm and tense vs. relaxed), which represents a low level of tense arousal (Thayer, 1990, 1997), and energetic arousal (tired vs. awake and without energy vs. full of energy).

#### Subjective Mental Demands

We further included typical situational demands of university students that are similar to the demands of high-knowledge workers. Our goal was to efficiently represent a broad spectrum of mental demands. Therefore, sensory, quantitative, and cognitive demands were included referring to a reference time frame of the preceding 2 hours. Oriented toward the German version (Nübling et al., 2005) of the Copenhagen Psychosocial Questionnaire (COPSOQ; Kristensen et al., 2002), sensory demands were captured with the item “. . . my activities required a high degree of concentration,” cognitive demands were surveyed with the item “. . . I had to be attentive to many things at the same time,” and quantitative demands were rated by the item “. . . I was under time pressure.” The three items were combined into one scale. The individual-level reliability estimate of these three items is .77 (Bonito et al., 2012), which can be considered respectable (Xie & De Vellis, 1992).

#### Objective Cognitive Performance

**Figure 1.**
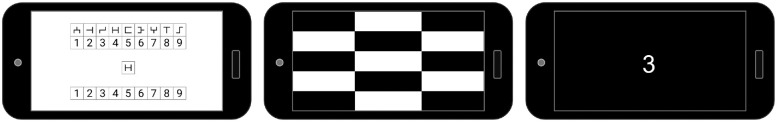
Screen Representations of the Digit Symbol Substitution Task, Psychomotor Vigilance Task, and Sustained Attention to Response Task.

Three cognitive tests with a total duration of approximately 3 minutes each were included in the study. The first task was a 180-second matching task adapted from the DSST (Boake, 2002; Wechsler, 1939). Throughout the task, random symbols appeared consecutively in the center of the screen (see Figure 1). The participant was asked to match these symbols to the correct digit in the bottom response bar with the help of the upper matching bar. The digit symbol assignment defined in a table on top of the screen varied with each measurement occasion. Response times and the number of errors were recorded.

In addition, a simple reaction time task similar to the PVT (Dinges & Powell, 1985) was presented in a 48-trial version. A black screen was initially shown, and the participants were asked to touch the screen as soon as a checkerboard pattern appeared (see Figure 1). The interstimulus interval varied from 2,000 ms to 5,000 ms. Reaction times were recorded and considered valid between 100 ms (Basner et al., 2011) and 30,000 ms.

Furthermore, a 135-trial Go-NoGo task similar to the SART (Robertson et al., 1997) was presented. In this task, digits from 1–9 appeared for 250 ms in a random order in the middle of the screen (see Figure 1). In between the digits, a black screen was shown for 900 ms. Reaction time and commission errors were recorded.

### Software and Hardware

All items were presented in the movisensXS application (movisens GmbH, Karlsruhe, Germany) on an Android 7.0 smartphone with a 5.0” display (1080 × 1920 pixels). Response times were recorded in milliseconds with one decimal. The cognitive tests were performed within the movisensXS smartphone application using the Presentation software (Version 18.0; Neurobehavioral Systems, Inc., Berkeley, CA, “www.neurobs.com”). We used the code provided by Neurobehavioral Systems and adapted it for smartphone use to program the cognitive tests.

### Data Analysis

We focused on six cumulative test segments from a duration of approximately 30 seconds to a duration of approximately 3 minutes for each test to answer the first research question on identifying the shortest measurement duration for each of the three test procedures that provides reliable measures of within-person change in typical cognitive performance. The programming of the DSST was not based on a fixed number of trials but on a fixed measurement duration of 180 seconds. Each test segment was conservatively set to 15 trials to prevent a >5% loss of DSST data sets for the comparative analysis. In the PVT, eight trials represent one test segment. As the original SART consists of 25 passes of digits 1–9 (Robertson et al., 1997), we tried to break down our approximately 3-minute SART—corresponding to 15 passes—into reasonable segments, although commission trials were presented randomly instead of being evenly spread. Therefore, we considered test segments of 3, 5, 8, 10, 13, and 15 passes, which corresponded to an average of 3, 5, 8, 10, 13, and 15 NoGo trials, respectively.

As the participants completed the smartphone questionnaires repeatedly over 14 consecutive days, the data show a hierarchical structure with trials nested within days nested within persons. Similar to the procedure of Sliwinski et al. (2018) and Brose et al. (2012), the reliability of change was analyzed by examining systematic within-person variation in the raw outcomes of cognitive performance in accordance with the procedure for diary studies recommended by Cranford et al. (2006) and Shrout and Lane (2012). Regarding the DSST, we focused on the reaction time and the number of errors. For the PVT, reaction time and the number of lapses, defined as reaction times exceeding 355 ms (Basner et al., 2011), were considered to calculate the reliability of day-to-day change. To evaluate the reliability of day-to-day change in the SART, we considered both reaction times in correct trials and commission errors. First, the SPSS (version 29) command VARCOMP was used to decompose the within-person variation into systematic variability (variation across occasions) and error (variation within occasions) for the outcomes considered for the different tests and test segments. For the SART, this was done separately for Go trials and NoGo trials, as these can be seen as indicators for different outcomes—Go trials measure the reaction times of correct trials, and NoGo trials measure commission errors. This led us to the six cumulative test lengths of 24, 40, 64, 80, 104, and 120 Go trials for reaction times and 3, 5, 8, 10, 13, and 15 commission trials. The reliability of day-to-day change was then estimated as recommended in equation (5) of Cranford et al. (2006) and equation (9) of Shrout and Lane (2012). Although, according to Nezlek (2017), the standards when interpreting within-person reliability may be less strict than the established standards for between-person reliability, we used the criteria proposed by Shrout (1998) as a conservative reference frame to interpret the reliability coefficients.

For further analyses, the shortest measurement duration that provided at least a fair reliability of change for errors (*R_c_* > 0.40) and at least a moderate reliability of change for reaction time (*R_c_* > 0.60) was selected for each test (Shrout, 1998). Based on the literature (Schmidt-Atzert et al., 2004) and in line with prior studies (e.g., T. Chung et al., 2020; Daniëls et al., 2020; Grant et al., 2017; Riedl et al., 2023), we focused on the most prominent outcomes for each test. For the DSST, we focused on the number of errors indicating response accuracy, and response efficiency was defined as the number of correct trials achieved within a pure reaction time of 1 second. This represents the often-used total number of correct trials within a predefined time span that we could not directly refer to due to the time-based instead of trial-based programming of the test. For the PVT, we considered lapses and mean reaction time. For the SART, we considered commission errors and the mean reaction time of correct trials.

To answer the second research question on the validity of the test versions with the previously defined minimum reliable measurement durations, we calculated within-person and between-person correlations of the person-mean and group-mean centered cognitive outcomes subjective concentration and difficulty in maintaining focused attention performance (Nezlek, 2017).

Multilevel models were built for further analyses to consider the dependency in the nested data set. The statistical analyses were performed with the SPSS command MIXED (version 29). First, null models were built to calculate the variance proportions at Level 1 (days) and Level 2 (participants) for the six different cumulative test segments. We created an increasing count variable separately for each test that we included as a covariate in the multilevel models to test for autocorrelations and linear practice-related trends, gaining information on practice effects due to increasing task experience.

We tested for multicollinearity of the predictor variables by calculating the variance inflation factor for all predictors and centered valence, tense arousal, energetic arousal, and mental demands on the person-mean. We then added the person-centered predictors to the multilevel models (Bryk & Raudenbush, 1992; Nezlek, 2011) to gain insight into the results linked to research question three on the relationship of situational cognitive outcomes with contextual time-variant variables. The linear trend remained in the model to account for practice effects and autocorrelations. Therefore, we specified time series multilevel models, including a fixed effect and a repeated effect for the count variable using restricted maximum likelihood estimation (Hox & McNeish, 2020). In addition, we specified a random intercept and added random slopes for all within-person variables. As recommended by Nezlek (2011), nonsignificant random slopes (*p* > .10) were removed stepwise to build parsimonious models (Bates et al., 2015).

## Results

### Reliability of Day-to-Day Change

All null models showed significant within-subject and between-subject variances, indicating the need for multilevel models. The intraclass correlations of the cognitive outcomes ranged from .16 (mean reaction time, 8-trial PVT) to .60 (mean reaction time of correct trials, 117- and 135-trial SART; see Table 1). Furthermore, the subjective variables showed fundamental proportions of within-subject variance (see Table 2).

**Table 1.** Intraclass Correlations for Different Objective Outcomes and Increasing Numbers of Cumulative Test Segments.

Objective outcome	Number of cumulative test segments					
	1	2	3	4	5	6
DSST errors	.19	.28	.31	.34	.38	.42
DSST response efficiency	.34	.45	.47	.50	.53	.53
PVT lapses	.37	.44	.46	.49	.50	.53
PVT mean reaction time	.16	.25	.28	.29	.27	.27
SART commission errors	.17	.24	.33	.41	.53	.59
SART mean reaction time of correct trials	.46	.49	.55	.57	.60	.60

*Note. N_2_* = 46, *N_1 DSST segment 1-3_* = 566, *N_1 DSST segment 4_* = 565, *N_1 DSST segment 5_* = 562, *N_1 DSST segment 6_* = 545, *N_1 PVT_* = 575, *N_1 SART_* = 559. One DSST test segment refers to 15 trials. One PVT test segment refers to eight trials. The cumulative SART test segments refer to 27, 45, 72, 90, 117, and 135 trials, respectively. DSST = Digit Symbol Substitution Task; PVT = Psychomotor Vigilance Task; SART = Sustained Attention to Response Task.

**Table 2.** Descriptive Statistics of the Study Variables for the 90-Second DSST, the 30-Second PVT, and the 90-Second SART.

Variable	*M*	*SD*_w_	*SD*_b_	*ICC*	1	2	3	4	5	6	7	8	9	10	11
1. DSST errors	1.84	1.80	1.36	.31		−.41**	.00	−.04	.07	−.03	−.06	.00	−.11**	−.12**	−.11*
2. DSST response efficiency	0.64	0.07	0.08	.47	−.33*		−.01	.06	−.01	.02	.10*	.07	.10*	.10*	.10*
3. PVT lapses	1.28	1.33	1.12	.37	.11	−.24		.33**	.03	−.03	.00	.04	−.01	−.08	−.03
4. PVT RT	314.72	190.70	98.76	.16	−.01	−.29	.80**		−.02	−.05	−.04	−.04	−.09*	.00	−.09*
5. SART commission errors	5.13	1.85	1.46	.33	.47**	−.16	.19	.13		−.33**	.05	−.05	.04	.00	−.00
6. SART RTC	285.30	42.79	50.08	.55	−.39**	−.04	.19	.17	−.69**		−.02	.03	−.05	−.10*	.01
7. Energetic arousal	3.97	1.14	0.61	.20	−.42**	.15	−.21	−.15	−.46**	.30*		.13**	.35**	.10**	.46**
8. Calmness	4.42	1.10	0.68	.26	−.35*	.16	−.12	−.07	−.23	.12	.61**		.57**	−.24**	.21**
9. Valence	4.65	1.07	0.67	.27	−.33*	.17	−.23	−.13	−.34*	.14	.65**	.81**		−.05*	.28**
10. Mental demands	2.34	1.42	0.71	.18	.20	−.19	.22	.22	.18	−.14	−.34*	−.40**	−.37*		.05*
11. Subjective concentration	1.28	1.13	0.59	.20	−.28	.05	−.02	.03	−.40**	.28	.71**	.63**	.61**	−.05	
12. Focused attention maintaining difficulty	1.67		0.59		.32*	−.30*	.07	.19	.35*	−.23	−.47**	−.27	−.38**	.20	−.37*

*Note. N_2_* = 46, *N_1 DSST_* = 566, *N_1 PVT_* = 575, *N_1 SART_* = 559, *N_1 subjective_* = 1,706. Above the diagonal, the within-person correlations are reported, and below the diagonal, between-person correlations based on aggregated data are shown. DSST = Digit Symbol Substitution Task; PVT = Psychomotor Vigilance Task; SART = Sustained Attention to Response Task; *SD*_w_ = within-subject standard deviation; *SD*_b_ = between-subject standard deviation; *ICC* = intraclass correlation; RT = reaction time; RTC = reaction time of correct trials.

* *p* < .05. **p < .01.

#### Digit Symbol Substitution Task

**Figure 2.**
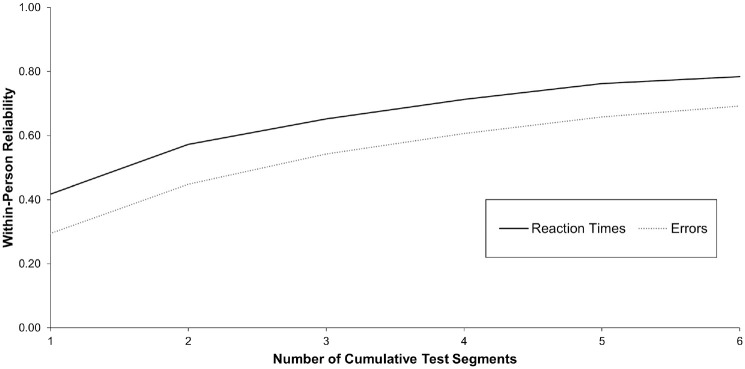
Within-Person Variability of Change for Cumulative Test Segments of the Digit Symbol Substitution Task. *Note.* One test segment refers to 15 trials.

Based on the criteria of Shrout (1998), errors made in the DSST reached fair reliability from a 60-second measurement duration onward (*R_c_*= 0.45) and moderate reliability from a 120-second measurement duration onward (*R_c_*= 0.61; see Figure 2). Reaction times showed a fair reliability from the 30-second measurement duration onward (*R_c_*= 0.42) and reached a moderate level from a 90-second measurement duration onward (*R_c_*= 0.65). Concerning RQ1, these results suggest that a 90-second version of the DSST might be sufficient to provide daily measures of cognitive performance with a fair to moderate reliability of change.

#### Psychomotor Vigilance Task

**Figure 3.**
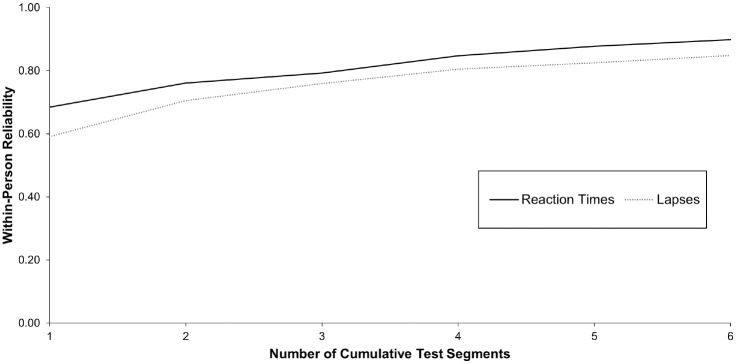
Within-Person Variability of Change for Cumulative Test Segments of the Psychomotor Vigilance Task. *Note.* One test segment refers to 8 trials. Lapses are defined as trials with reaction times exceeding 355 ms.

Reaction times in the PVT showed moderate reliability of change from a 30-second measurement duration onward (*R_c_*= 0.68), reaching a substantial level as of a 2-minute measurement duration (*R_c_*= 0.85; see Figure 3). The reliability of day-to-day change in lapses was fair for the 30-second PVT (*R_c_*= 0.59) and reached a moderate level from a 60-second measurement duration onward (*R_c_*= 0.71). Based on these results and referring to our predefined criteria, it can be concluded that a measurement duration of 30 seconds might already assure a sufficient reliability of day-to-day change in reaction time and lapses (RQ1).

#### Sustained Attention to Response Task

**Figure 4.**
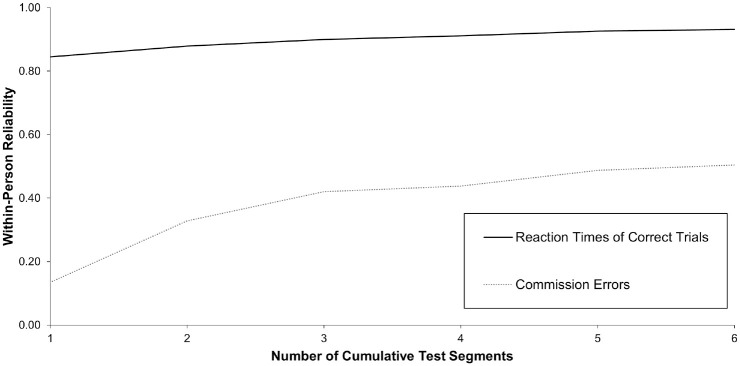
Within-Person Variability of Change for Cumulative Test Segments of the Sustained Attention to Response Task. *Note.* The cumulative test segments refer to 24, 40, 64, 80, 104, and 120 Go trials for reaction times of correct trials and 3, 5, 8, 10, 13, and 15 NoGo trials for commission errors.

Surprisingly, reaction times of correct trials in the SART already showed substantial reliability of change from a 30-second measurement duration onward (*R_c_*= 0.84; see Figure 4). Commission errors reached fair reliability of change from eight NoGo trials onward (*R_c_*= 0.42), corresponding to a 90-second measurement duration. Answering RQ1, these results indicate that the SART with a duration of 90 seconds might provide the optimal compromise of efficient data collection and satisfying reliability of day-to-day change.

### Validity and Practice-Related Improvement

The variation in test performance across the assessment period is descriptively presented in the supplements (see Supplemental Table S1).

#### Digit Symbol Substitution Task

In the 90-second DSST, which covers 45 trials, participants made on average *M* = 1.84 (*SD* = 2.26) errors, which resulted in a mean response efficiency of *M* = 0.64 (*SD* = 0.11) correct trials achieved within a pure reaction time of 1 second (see Table 2). Between-person, participants who reported difficulties in maintaining focused attention made, on average, more errors in the DSST (*r* = .32) and conducted the test less efficiently (*r* = −.30). Furthermore, within-person, DSST performance was significantly correlated with subjective concentration (see Table 2), supporting construct validity (RQ2).

While the number of errors in the DSST remained constant (*γ =* 0.03, *SE* = 0.02, *p* = .21), an increase in response efficiency (*γ =* 0.01, *SE* = 0.00, *p* < .01) was observed with accumulating task experience, indicating a practice-related performance improvement (see Table 3). Autocorrelations were not significant for the number of errors (*r* = .03; *p* = .60) or for response efficiency (*r* = .04; *p* = .48).

**Table 3.** Results From Hierarchical Linear Modeling to Predict Digit Symbol Substitution Task (DSST) Performance.

	DSST errors		DSST Response efficiency	
	Model 1	Model 2	Model 1	Model 2
	Estimate (*SE*)	Estimate (*SE*)	Estimate (*SE*)	Estimate (*SE*)
Fixed effects				
Intercept	1.633 (0.251)**	1.579 (0.244)**	0.598 (0.013)**	0.598 (0.013)**
Task experience	0.028 (0.022)	0.034 (0.021)	0.006 (0.001)**	0.006 (0.001)**
Energetic arousal		−0.009 (0.073)		0.004 (0.003)
Calmness		0.084 (0.127)		0.002 (0.004)
Valence		−0.230 (0.115)*		0.004 (0.004)
Mental demands		−0.103 (0.058)		0.006 (0.002)*
Random effects				
Energetic arousal				
Calmness		0.330 (0.140)*		
Valence		0.165 (0.092)		
Mental demands				
Level 1 residual variance	3.513 (0.221)**	2.936 (0.201)**	0.006 (0.000)**	0.005 (0.000)**
Autocorrelation parameter	0.028 (0.052)	0.014 (0.054)	0.036 (0.050)	0.041 (0.051)
Level 2 residual variance	1.587 (0.398)**	1.555 (0.383)**	0.005 (0.001)**	0.005 (0.001)**

*Note. N_2_* = 46 (between person), *N_1_* = 566 (within person). DSST = Digit Symbol Substitution Task; *SE* = standard error.

* *p* < .05. **p < .01.

#### Psychomotor Vigilance Task

In the 30-second PVT, comprising eight trials, participants reacted on average within *M* = 314.76 ms (*SD* = 217.19) to the stimulus and caused on average *M* = 1.28 (*SD* = 1.76) lapses. Within-person, subjective concentration correlated negatively with the PVT mean reaction time (*r* = −.09), supporting construct validity (RQ2). No significant between-person correlations of PVT performance with subjective concentration or difficulty in maintaining focused attention were found (see Table 2).

While the number of lapses increased (*γ =* 0.07, *SE* = 0.02, *p* < .01), the mean reaction time remained stable with increasing task experience (*γ =* 0.66, *SE* = 2.21, *p* = .77), indicating a performance decrease over time (see Table 4). Autocorrelations were significant for the number of lapses (*r* = .11, *p* < .05) but not for mean reaction time (*r* = −.01, *p* = .89).

**Table 4.** Results From Hierarchical Linear Modeling to Predict Psychomotor Vigilance Task (PVT) Performance.

	PVT lapses		PVT reaction time	
	Model 1	Model 2	Model 1	Model 2
	Estimate (*SE*)	Estimate (*SE*)	Estimate (*SE*)	Estimate (*SE*)
Fixed effects				
Intercept	0.791 (0.202)**	0.798 (0.202)**	308.639 (21.353)**	307.398 (19.282)**
Task experience	0.069 (0.017)**	0.068 (0.017)**	0.659 (2.210)	0.544 2 (0.121)
Energetic arousal		0.009 (0.054)		−4.663 (7.737)
Calmness		0.021 (0.067)		5.021 (9.572)
Valence		−0.044 (0.069)		−14.380 (13.232)
Mental demands		−0.058 (0.042)		0.125 (5.912)
Random effects				
Energetic arousal				
Calmness				
Valence				3188.937 (1028.683)**
Mental demands				
Level 1 residual variance	1.883 (0.121)**	1.883 (0.121)**	39373.542 (2432.695)**	35052.946 (2206.695)**
Autocorrelation parameter	0.108 (0.049)*	0.100 (0.049)*	−0.012 (0.088)	−0.020 (0.073)
Level 2 residual variance	1.097 (0.268)**	1.097 (0.268)**	7430.886 (2167.462)**	4787.433 (1582.640)**

*Note. N_2_* = 46 (between person), *N_1_* = 575 (within person). PVT = Psychomotor Vigilance Task; *SE* = standard error.

* *p* < .05, **p < .01.

#### Sustained Attention to Response Task

In the 90-second SART, which includes 72 trials, on average, *M* = 7.88 (*SD* = 1.76) NoGo trials were presented, resulting in *M* = 5.13 (*SD* = 2.34) commission errors on average. The mean reaction time of correct trials was *M* = 285.30 ms (*SD* = 66.90). Between person, the number of commission errors in the SART correlated significantly with subjective concentration (*r* = −.40) and difficulty in maintaining focused attention (*r* = .35), supporting construct validity (RQ2). No significant within-person correlations were found for SART performance with subjective concentration (see Table 2).

The number of commission errors remained stable with increasing task experience (*γ =* 0.04, *SE* = 0.02, *p* = .08), while the mean reaction time of correct trials decreased (*γ =*−1.46, *SE* = 0.65, *p* < .05), indicating a performance improvement with increasing practice (see Table 5). Autocorrelations were significant for the mean reaction time of correct trials (*r* = .27, *p* < .01) but not the number of commission errors (*r* = .06, *p* = .25).

**Table 5.** Results From Hierarchical Linear Modeling to Predict Sustained Attention to Response Task (SART) Performance.

	SART commission errors		SART reaction time of correct trials	
	Model 1	Model 2	Model 1	Model 2
	Estimate (*SE*)	Estimate (*SE*)	Estimate (*SE*)	Estimate (*SE*)
Fixed effects				
Intercept	4.844 (0.264)**	4.821 (0.267)**	296.147 (8.619)**	296.593 (8.627)**
Task experience	0.042 (0.023)	0.046 (0.023)	−1.463 (0.647)*	−1.535 (0.650)*
Energetic arousal		0.092 (0.100)		−0.322 (1.797)
Calmness		−0.214 (0.092)*		2.182 (2.043)
Valence		0.178 (0.100)		−4.667 (2.210)*
Mental demands		−0.040 (0.060)		−2.772 (1.353)*
Random effects				
Energetic arousal		0.148 (.081)		
Calmness				
Valence				
Mental demands				
Level 1 residual variance	3.747 (0.240)**	3.524 (0.235)**	2108.380 (157.213)**	2092.363 (157.479)**
Autocorrelation parameter	0.057 (0.049)	0.058 (0.051)	0.270 (0.054)**	0.276 (0.054)**
Level 2 residual variance	1.756 (0.449)**	1.829 (0.462)**	2283.796 (541.043)**	2280.224 (540.919)**

*Note. N_2_* = 46 (between person), *N_1_* = 559 (within person). *SE* = standard error.

* *p* < .05. **p < .01.

### Associations With Contextual Time-Variant Variables

With all Variance Inflation Factors smaller than 1.67, preliminary analyses indicated no risk of multicollinearity for the predictors in the multilevel models (Tabachnick & Fidell, 2013).

#### Digit Symbol Substitution Task

In the 90-second DSST, when controlling for task experience and the influence of the other predictors, fewer errors were made when participants reported a more positively pronounced valence (*γ =*−0.23, *SE* = 0.11, *p* < .05; see Table 3). Furthermore, participants completed the DSST more efficiently when the preceding mental demands were higher than usual (*γ =* 0.01, *SE* = 0.00, *p* < .05). To conclude, valence and prior mental demands were positively related to DSST performance. For energetic arousal and calmness, however, no significant effects were observed (see Table 3).

#### Psychomotor Vigilance Task

Within person, none of the main effects were significant for the 30-second PVT when controlling for practice effects and the influence of the other predictors (see Table 4).

#### Sustained Attention to Response Task

The observed mean reaction times of correct trials in the 90-second SART were faster when the participants reported increased valence (*γ =*−4.67, *SE* = 2.21, *p* < .05) and when preceding mental demands were higher (*γ =*−2.77, *SE* = 1.35, *p* < .05; see Table 5). Furthermore, calmness is related to a reduced number of commission errors (*γ =*−0.21, *SE* = 0.09, *p* < .05). Energetic arousal is not significantly related to momentary performance in the 90-second SART (see Table 5).

## Discussion

The central aim of our study was to provide evidence-based recommendations concerning the ambulatory use of brief smartphone-based versions of established cognitive test procedures in healthy adult populations. The challenge is to keep the measurement duration as short as possible while still assuring data quality. Therefore, we identified the minimum measurement duration of these test procedures that can provide reliable state measures of cognitive performance (RQ1). Subsequently, we evaluated the validity of these brief cognitive measures (RQ2). Furthermore, by identifying significant within-person associations with contextual time-variant variables, namely, valence, energetic arousal, calmness, and mental demands, we aimed to provide an outlook on possible fruitful fields of application for the respective tests (RQ3).

Based on predefined criteria (Shrout, 1998), the 30-second PVT and the 90-second DSST and SART provided the best compromise of brief and reliable measurements. This answers our first research question (RQ1), indicating that the tests in the mentioned measurement durations provide reliable smartphone-based measures of typical performance in field studies with healthy adult populations. Overall, the reliability coefficients of the tests in the chosen lengths are comparable to those of similar tests in former studies (e.g., Sliwinski et al., 2018).

Furthermore, all three cognitive test procedures correlated significantly with subjective cognitive outcomes, supporting the construct validity of the brief test versions (RQ2). While both DSST outcomes and the mean reaction time in the PVT were situationally correlated with subjective concentration, we did not find within-person correlations for the SART outcomes. Nonetheless, commission errors made in the SART were correlated with concentration on a between-person level. In addition, the between-person correlation of the difficulty in maintaining focused attention with the DSST outcomes further supports construct validity. No significant correlations were found for the number of lapses in the PVT, potentially because 355 ms might not be the threshold that provides optimal sensitivity for the very brief 30-second measurement duration (Basner et al., 2011).

The participants completed the 90-second DSST more efficiently with increasing task experience, indicating a practice-related improvement. This is in line with the results of previous studies (Beres & Baron, 1981; van Oirschot et al., 2020; Verhagen et al., 2019) and therefore further supports construct validity. For the 30-second PVT, we observed a significantly increasing number of lapses over the study period, potentially indicating a lack of motivation, suggesting that this task might be less pleasant than the other tasks, likely resulting from its monotony. With increasing task experience, the 90-second SART was completed faster, whereas the number of commission errors did not significantly increase, overall indicating increasing task proficiency.

Providing evidence for RQ3 concerning mood and in line with the results of Verhagen et al. (2019), momentary valence was significantly associated with an increased performance in the 90-second DSST, as indicated by a reduced number of errors. For the 90-second SART, valence significantly predicted faster mean reaction times of correct trials but not a reduced number of commission errors, which is only partly in line with the results of Brinker et al. (2013), who found that mood significantly correlated with the number of commission errors in Go-NoGo tasks with an interstimulus interval of 1 second. However, the NoGo proportion of the test used in their study was much larger than that used in ours (4 out of 9 vs. 1 out of 9), and the NoGo stimuli varied, while the Go stimulus remained consistent. As the mood effects in this study did not appear for all levels of task difficulty, the different design of our test might explain the absent effect of valence on the number of commission errors in our study. The results for the 90-second smartphone-based DSST and SART reflect that valence plays a crucial role in the first stages of information processing (Fredrickson & Branigan, 2005), supporting the construct validity of these two tasks (RQ2) and indicating that they might be profitable test procedures for application in ambulatory mood research (RQ3).

Concerning momentary levels of energetic arousal, significant within-person correlations were detected for response efficiency in the DSST. However, these correlations did not remain significant when controlling for practice effects and further relevant context variables in the multilevel models. One possible explanation for the absent association of the objective performance indicators with energetic arousal might be that according to G. Matthews et al. (1990), the performance facilitating effect becomes especially evident for tasks with high task difficulty. Our tasks might not be difficult enough to reflect a certain effect, especially due to the reduced measurement duration. Our healthy sample and the measurement period during daytime hours might also play a crucial role, as the variability in energy levels and tiredness might not have been large enough to show a crucial effect on cognitive performance. However, these results are especially surprising for the PVT, which has thus far mainly been applied in the context of sleep deprivation, contradicting the significant correlation of subjective fatigue and reaction time in the PVT in the study of Price et al. (2017). Nevertheless, the crucial difference might be that in their study, the PVT was presented every day at the same time and correlated with subjective fatigue at the day level instead of at the momentary level, as in our study. Furthermore, the construct of fatigue used in their study was of a very broad nature, covering fatigue in general and a lack of initiative or concentration difficulties. In addition, in other studies, shorter PVT measurement durations were associated with a lower sensitivity to sleepiness (Loh et al., 2004; Roach et al., 2006). Given the limited evidence of sensitivity to changes in energetic arousal, we cannot generally recommend the application of shortened test procedures in studies related to energetic arousal in healthy samples (RQ3). However, the potentially fruitful application of brief tests in the context of time-constrained workplaces that are, for example, linked to shift work causing a larger variability in energetic arousal should be further tested.

In line with the ACT (Eysenck et al., 2007) and former studies that used the SART in recovery research (Pasanen et al., 2018; Riedl et al., 2023), calmness was significantly associated with a reduced number of commission errors in the SART, which indicates enhanced cognitive inhibition. Sliwinski et al. (2006) noted that stress effects do not become evident in simple tasks, such as the PVT. Resource depletion due to stress impairs cognitive processing when it is effortful (Kahneman, 1973; Oei et al., 2006), which might be especially the case for the Go-NoGo SART, which requires top-down control (Sänger et al., 2014; Shields et al., 2016). In addition, the ACT states that momentary stress especially impedes cognitive control (Eysenck et al., 2007) and central executive functions of inhibition (Miyake et al., 2000). This can explain why no effects of tense arousal were found for the DSST and PVT and further supports the construct validity of the ambulatory SART (RQ2). To conclude, the ambulatory 90-second SART might be a promising test procedure for application in real-life stress and recovery research (RQ3).

In line with the assumptions of the MART and the results of Liao and Moray (1993), preceding mental demands in the 2 hours prior to the objective cognitive assessment were significantly associated with momentary DSST efficiency as well as the mean reaction time of correct trials in the SART. These results support the validity of the 90-second DSST and SART (RQ2) and further indicate that they can be fruitfully applied to learn more about demand-induced effects on mental performance (RQ3). The lack of significant effects of preceding mental demands on PVT performance may be because the PVT is more sensitive to fatigue than to cognitive stimulation. However, our study design did not allow for the detection of distally related strain effects (Meijman & Mulder, 1998), as our reference frame for the mental demands was the preceding 2 hours, and two-thirds of the measurements took place in the morning or early afternoon.

### Practical Recommendations

Overall, due to convincing reliability (RQ1) and validity evidence (RQ2), we recommend the smartphone-based 90-second SART and DSST as well as the 30-second PVT for the repeated assessment of typical cognitive functioning in healthy samples in the real-life context. Furthermore, our results indicate that the brief DSST and SART are meaningful for identifying the relationship between cognitive performance and contextual time-variant variables, such as mood and preceding mental demands, while the 90-second SART appears additionally sensitive to changes in subjectively perceived calmness (RQ3). This leads us to the assumption that both tests may be fruitfully applied in the context of mood research and gainful in clarifying research questions regarding the performance-enhancing effects of mental demands. In addition, a potential fruitful application of the SART in ambulatory field studies related to stress and recovery can be assumed due to the relationship of the number of commission errors with calmness or tense arousal. We provide the 90-second DSST (“http://www.neurobs.com/ex_files/expt_view?id=300”) and SART (“https://www.neurobs.com/ex_files/expt_view?id=301”) for free in the experiment archives of Neurobs Presentation to facilitate its use by other researchers.

Both tests appear appropriate and fruitful to deepen the knowledge on concomitants of mental demands for typical cognitive performance. In particular, the number of errors in the DSST and the mean reaction time of correct trials in the SART appear to be sensitive in this regard. In addition, the number of correct trials in the DSST and the mean reaction time of correct trials in the SART appear suited to picture the effects of mood on typical cognitive performance. Furthermore, the number of commission errors in the SART appears sensitive to calmness and therefore a promising outcome for ambulatory workplace stress and recovery studies in which objective outcomes are thus far strongly underrepresented (Sonnentag et al., 2017). However, the final choice of the outcome and task should always be guided by the cognitive functions to be investigated. While the DSST can be seen as an indicator of general cognitive functioning (Dickinson et al., 2007; Salthouse, 1996) that has low specificity (Amaresha et al., 2014; Sandry et al., 2021), the SART provides more specific information about controlled attention (Manly et al., 2003), cognitive inhibition (Johnson et al., 2007), and attentional lapses (Manly et al., 2003; Robertson et al., 1997; Smilek et al., 2010).

### Limitations and Future Directions

Our aim was to test the reliability and validity of brief smartphone-based ambulatory cognitive tests in a healthy sample and to gain information about in which research contexts of related time-variant variables their application might be profitable. Our sample, however, included mainly female psychology students in their early twenties, which reduces the generalizability to other healthy populations. Since access to psychology studies in Germany is strictly regulated by numerus clausus, it can be assumed that the IQ of the sample was above the average IQ of the overall population (Gut et al., 2012). Furthermore, mental demands represent the typical demands of university students or high-knowledge workers. Although we tried to represent a broad spectrum of mental demands by including sensory, quantitative, and cognitive demands, there was a shortcoming of typical demands of other occupational groups, such as emotional demands. Therefore, brief test procedures should be further evaluated in different professional contexts with more heterogeneous samples.

One methodological issue is that splitting the tests into cumulative test segments was quite artificial. For practical reasons, we chose a total measurement duration of approximately 3 minutes. This led to the problem that, for the SART, the six-test segments were of unequal length, as the 135 trials could not be reasonably split evenly. Furthermore, the commission trials were randomly spread. Even though the NoGo proportion referred on average to the foreseen proportion for the cumulative test segments, random deviations were possible. In addition, the programming of the DSST was not based on a fixed number of trials but on the temporal test duration. Due to a lack of temporal information in the output files, we conservatively split the test segments based on practical considerations. Since the participants had performed different numbers of trials in the predefined total measurement interval, the measurement segments defined based on a certain number of trials correspond to individually varying periods. Furthermore, we could not directly evaluate the often-used outcome of total trials achieved within a fixed period (e.g., T. Chung et al., 2020). However, the response efficiency should be a closely related outcome that can be considered equivalent. Further evidence is needed to determine whether the 90-second version of the DSST can replicate our results, especially considering the total number of correct trials.

A potential loss of motivation of the participants to repeatedly perform the 3-minute PVT in our study highlights the need to apply shorter versions of established test procedures in ambulatory assessment studies. Even though the 30-second PVT shows satisfying reliability of change and significant correlations support its validity, we could not identify significant associations with contextual time-variant study variables. Therefore, the shortened version should be further tested in other study designs, for example, after prolonged exposure to demands in time-constrained work environments, to demonstrate its fruitful application in ambulatory assessment studies with healthy adults. In addition, a potential adjustment of the lapse threshold should be investigated.

As previous studies indicate that cognitive performance differs throughout the day due to circadian variation (e.g., Gaggero & Tommasi, 2023), time-of-day effects on cognitive performance could further support the construct validity of our brief tests. However, as we presented each test only once per day, our study design did not allow us to calculate performance trajectories throughout the day. Future research should take advantage of our shortened smartphone-based test procedures by assessing cognitive performance repeatedly throughout the day, for example, every 2 hours (Kosenkranius et al., 2023). Such research designs would not only generate more concrete insights about circadian variation in cognitive performance outcomes but also further support the construct validity of the shortened ambulatory cognitive assessments.

In our study, we focused primarily on within-person reliability and momentary associations with time-variant variables, aiming for the use of these brief smartphone-based test procedures in ambulatory assessment research focusing on situational relationships. However, to provide a further outlook, these tests might also be profitable between-person measures. If the aim is to assess typical performance, aggregated repeated within-person measures of cognitive functioning conducted in real-life settings under natural conditions might provide a more accurate estimate than one-time measures in controlled laboratory settings (Moore et al., 2016). This practical use requires further investigation, which in turn can also profoundly supplement the validation of our shortened ambulatory test procedures. Even though concentration is closely related to attention (Matlin, 2009; Moran, 2012), it might be slightly short-sighted to base the construct validity of objective cognitive measures primarily on the correlation with subjective concentration. Individuals do not have a good sense in subjectively judging abstract constructs such as concentration (Bermudez et al., 2016; Cote & Buckley, 1987; Dorrian et al., 2003), which leads to weak correlations with objectively measured cognitive performance (Freund & Kasten, 2012). Thus, demonstrating strong correlations of the mean score of our repeated shortened smartphone-based tests with traditional laboratory test scores might further support the validity of our abbreviated instruments. Generally, even though the assessment of cognitive performance in real-life situations can add valuable insights into typical performance (Allard et al., 2014; Moore et al., 2017; Timmers et al., 2014), great care is needed to avoid assessment biases, for example, by carefully instructing participants to reduce distractions in assessment situations.

## Conclusion

Our results indicate that the smartphone-based 90-second DSST and SART as well as the 30-second PVT provide efficient, reliable, and valid state measures of typical cognitive performance in real-life settings. While the DSST and SART appear especially profitable in the context of mood research and for research questions regarding preceding mental demands, the SART appears to additionally provide a fruitful objective indicator for stress and recovery research. We hope our research encourages others to include objective cognitive measures in their ambulatory field studies.

## Supplemental Material

sj-docx-1-asm-10.1177_10731911231213845 – Supplemental material for Measuring Situational Cognitive Performance in the Wild: A Psychometric Evaluation of Three Brief Smartphone-Based Test Procedures Supplemental material, sj-docx-1-asm-10.1177_10731911231213845 for Measuring Situational Cognitive Performance in the Wild: A Psychometric Evaluation of Three Brief Smartphone-Based Test Procedures by Johanna Perzl, Elisabeth Maria Riedl and Joachim Thomas in Assessment
