# Supplementary material for: Measuring Situational Cognitive Performance in the Wild: A Psychometric Evaluation of Three Brief Smartphone-Based Test Procedures
Source: Assessment. 2023 Dec 14;31(6):1270–91. doi: 10.1177/10731911231213845 (PMC11292980; doi:10.1177/10731911231213845)
Supplement: sj-docx-1-asm-10.1177_10731911231213845 – Supplemental material for Measuring Situational Cognitive Performance in the Wild: A Psychometric Evaluation of Three Brief Smartphone-Based Test Procedures [file sj-docx-1-asm-10.1177_10731911231213845.docx]

**Table S1**

Variation in Test Performance across the Assessment Period

| Variable | Day 1 | | Day 2 | | Day 3 | | Day 4 | | Day 5 | | Day 6 | | Day 7 | | Day 8 | | Day 9 | | Day 10 | | Day 11 | | Day 12 | | Day 13 | | Day 14 | |
| --- | --- | --- | --- | --- | --- | --- | --- | --- | --- | --- | --- | --- | --- | --- | --- | --- | --- | --- | --- | --- | --- | --- | --- | --- | --- | --- | --- | --- |
| 1. DSST errors | 1. | 29 | 2. | 15 | 1. | 64 | 1. | 93 | 1. | 95 | 1. | 90 | 1. | 66 | 1. | 60 | 1. | 88 | 1. | 53 | 1. | 88 | 2. | 19 | 1. | 92 | 2. | 07 |
| 2. DSST response efficiency | 0. | 59 | 0. | 61 | 0. | 61 | 0. | 61 | 0. | 64 | 0. | 65 | 0. | 66 | 0. | 65 | 0. | 66 | 0. | 67 | 0. | 67 | 0. | 66 | 0. | 66 | 0. | 67 |
| 3.PVT lapses | 0. | 87 | 0. | 88 | 0. | 76 | 0. | 86 | 1. | 05 | 1. | 39 | 1. | 41 | 1. | 26 | 1. | 50 | 1. | 83 | 1. | 61 | 1. | 44 | 1. | 35 | 1. | 69 |
| 4. PVT RT | 396. | 91 | 290. | 42 | 279. | 54 | 282. | 66 | 283. | 28 | 315. | 11 | 306. | 77 | 304. | 47 | 328. | 29 | 325. | 15 | 347. | 89 | 314. | 56 | 313. | 41 | 318. | 43 |
| 5. SART commission errors | 4. | 62 | 4. | 63 | 5. | 03 | 5. | 16 | 5. | 41 | 4. | 62 | 5. | 03 | 5. | 49 | 5. | 05 | 5. | 80 | 5. | 63 | 4. | 76 | 5. | 24 | 5. | 34 |
| 6. SART RTC | 312. | 84 | 296. | 47 | 278. | 12 | 279. | 81 | 285. | 91 | 287. | 00 | 283. | 25 | 276. | 16 | 280. | 96 | 274. | 68 | 282. | 26 | 287. | 44 | 266. | 64 | 303. | 81 |

*Note.* *N* = 36-44. DSST = Digit Symbol Substitution Task. PVT = Psychomotor Vigilance Task. SART = Sustained Attention to Response Task. RT = reaction time. RTC = reaction time of correct trials.
